# Impact of plant fiber reinforcement on mechanical properties of PMMA denture base resin: a systematic review

**DOI:** 10.2340/biid.v13.45632

**Published:** 2026-03-30

**Authors:** Muhammad Khawaja Hammad Uddin, Nazrah Mehar, Muhammad Ammar Khan, Muhammad Hassan Khoso, Bilquess Saba, Muneeb Ahmed Lone, Ayesha Akram, Syeda Mamoona Mateen, Muhammad Amber Fareed, Muhammad Sohail Zafar

**Affiliations:** a Department of Science of Dental Materials, Dr. Ishrat-ul-Ebad Khan Institute of Oral Health Sciences; b School of Dental Care Professionals, Dow University of Health Sciences, Karachi, Pakistan; c Department of Oral Biology, Dr. Ishrat-ul-Ebad Khan Institute of Oral Health Sciences, Dow University of Health Sciences, Karachi, Pakistan; d Department of Medicine, Ziauddin Medical College, Ziauddin University, Karachi, Pakistan; e Department of Prosthodontics, Dr. Ishrat-ul-Ebad Khan Institute of Oral Health Sciences, Dow University of Health Sciences, Karachi, Pakistan; f Bahria Medical and Dental College, Bahria University of Health Sciences, Karachi, Pakistan; g Dr. Panjwani Center for Molecular Medicine and Drug Research, International Center for Chemical and Biological Sciences, University of Kar achi, Karachi, Pakistan; h Department of Clinical Sciences, College of Dentistry, Ajman University, Ajman, United Arab Emirates; i Centre of Medical and Bio- allied Health Sciences Research, Ajman University, Ajman, United Arab Emirates; j School of Dentistry, University of Jordan, Amman, Jordan

**Keywords:** natural fibers, plant fibers, denture base materials, biomaterials

## Abstract

**Introduction:**

To improve the clinical outcomes and long-term durability of polymethyl methacrylate (PMMA) denture-based resins, they have undergone several developments and changes in their chemical composition to enhance their mechanical properties, including reinforcing them with natural fibers.

**Aim:**

This systematic review aimed to explore the influence of plant fiber reinforcement on the mechanical characteristics of PMMA denture resins.

**Material and methods:**

For this purpose, the formulated focus question was ‘Does incorporating plant fibers in PMMA denture resins improve their mechanical strength?’ Using the PRISMA protocol, three electronic databases (Google Scholar, Pub Med, and Science Direct) were searched using the specific keywords: (plant fibers, natural fibers, and PMMA denture base resins) between 2014 and 2024. After evaluating the papers against the inclusion criteria, only those that met all requirements were included in the study. Additionally, all articles were screened for quality assessment, with particular attention to potential bias arising from experimental design and methodological reporting.

**Results:**

This review included eight experimental laboratory-based studies that met the inclusion criteria. All studies had low risk of bias, except one, which had moderate risk of bias. The evaluation of reinforced resins’ flexural strength was a noticeable trend observed in studies, with the maximum weight percentage utilized in the studies being 10 wt%. While flexural strength was primarily assessed by the majority of studies, other mechanical properties – including hardness, impact strength, tensile strength, and compressive strength – were also evaluated.

**Conclusions:**

The analysis revealed that the flexural strength and surface hardness of PMMA denture resins were significantly enhanced through the addition of plant fiber reinforcement. However, limited and inconclusive data are present for other mechanical properties (impact, compressive, and tensile strength). Extended experimental studies related to aging and various storage protocols, as well as clinical trials, are essential to determine its clinical relevance and longevity.

## Graphical abstract

**Figure:**
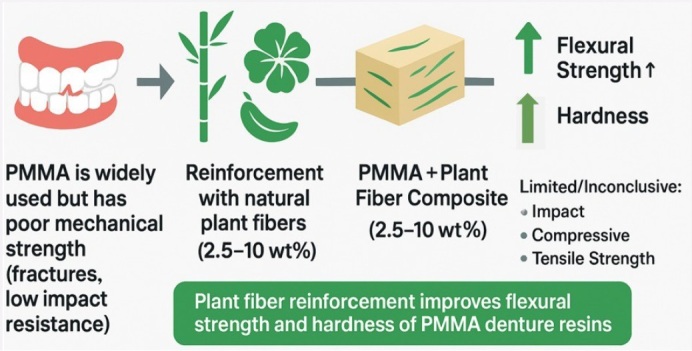
GRAPHICAL ABSTRACT

**KEY MESSAGES**

- The addition of plant fibers to polymethyl methacrylate (PMMA) denture base resins greatly improves their flexural strength and hardness, enhancing the resistance of the material to fracture and deformation.
- The reinforcing action of plant fibers is a function of fiber type, concentration, and surface treatment, with maximum improvements generally being obtained at lower weight percentages and with treated fibers.
- Existing evidence for other mechanical properties, including impact, tensile, and compressive strength, is restricted and inconclusive, highlighting the need for further investigations.

## Introduction

Polymers, an emerging and versatile class of biomaterials, have been frequently employed in various practical applications in the dental setting [1, 2]. Among polymers, polymethyl methacrylate (PMMA) has received remarkable attention and widespread use across various dental settings including dental laboratories (for the fabrication of post-treatment dental retainers and prosthesis and for repairing or reconstructing them as well), dental practice (for refurbishing dentures and temporary restorations), and the commercial sector (designing and manufacturing artificial teeth) [3]. PMMA is primarily employed for constructing dental prostheses due to several advantages, including affordability, biocompatibility, convenient and straightforward processing, stability in the oral cavity, and esthetically pleasing results. Despite these benefits, its poor mechanical and physical properties render it unsuitable for being regarded as an ideal denture base material [4]. However, ongoing research aims to improve their mechanical properties and address limitations like microbial colonization [5]. Numerous studies have been conducted to overcome these limitations by using various curing techniques or by incorporating different fillers, chemicals, and fibers in their composition [6–8]. The flexural, impact, and fatigue resistance of acrylic resin have all been improved using various fibers reinforcements. Until now, a variety of fibers, including nylon, polyethylene, polyamide fiber, and glass fibers, have been integrated with PMMA to strengthen its physico-mechanical properties [9, 10].

Scientists have also investigated various natural fibers due to their several benefits, including renewability, biodegradability, low cost, good strength-to-weight ratio, reduced density per unit volume, corrosion-resistance, and sustainable weight-specific strength [5, 11–13]. In contrast to synthetic fibers, natural fibers are cheaper and frequently available and less hazardous in relation to human health and the environment. Natural fibers are obtained from a diverse range of plant and animal sources [4]. Animal-sourced fibers are wool, silk, and feathers from chickens [14]. Plant fibers include sisal (*Agave sisalana*), hemp (*Cannabis sativa*), bamboo, coir (the husk of a coconut), flax (*Linum usitatissimum*), kenaf (*Hibiscus cannabinus*), jute, ramie, oil palm, pineapple, banana, and cotton [14, 15]. Since ancient times, people have used herbs and plants to treat a various range of illnesses. Their therapeutic and medicinal value in preventing and treating various pathologies has been established. Plant fibers have demonstrated various biological and medicinal benefits, particularly byproducts obtained from plants. They can provide anti-microbial, fungal-resistant, analgesic, anti-inflammatory, regenerative, and anti-tumor properties [16]. Consequently, plant fibers have been incorporated in a variety of dental biomaterials such as denture bases [17], dental implants [18], restorative materials [19], and mouthwashes [20]. They have also been utilized for tissue engineering, including stem cell studies [21]. Plant fibers have demonstrated a significant antimicrobial effect against periodontal pathogens and cariogenic bacteria [22, 23]. Plant extract can also enhance the anti-microbial and mechanical strength of dental implants, and also achieve chemical stability and biocompatibility [24]. Plant fiber-incorporated scaffold can enhance osteodifferentiation and mineralization of human dental stem cells (hDPSCs), which makes it a favorable candidate to be used in bone tissue engineering. [21]. Plant fibers have demonstrated anti-carcinogenic effect, enamel remineralization, angiogenesis, odontogenic differentiation effects [25–30] and wound healing properties [31–33].

Research has shown that the incorporation of plant fibers in denture base resins decreases their thermal conductivity and increases water sorption and water solubility [34]. Increased water sorption and solubility accelerate the biodegradation process of plant fibers [35]. Chemical treatments such as alkali treatment, silanization, and acetylation have been shown to remove unstable amorphous components, reduce hydrophilicity, and create a more durable interface within polymer composites [35, 36]. Plant fibers have also been used as a plasticizer in place of dibutyl phthalate due to its non-cytotoxicity and a lack of ester leaching [37]. Antimicrobial capability of plant fibers incorporated in PMMA denture resins has been evaluated, and it has shown a promising effect against *Candida albicans*, which is mainly responsible for denture-associated fungal infections [38–40].

The rationale behind employing plant fibers as reinforcers in PMMA is due to the fact that PMMA has mechanical weaknesses, which include low flexural strength and a lack of impact resistance. Plant fibers are also an excellent solution to the problem, being both sustainable and economical, with a high strength/weight ratio, and increased biocompatibility. Moreover, they can enhance such important mechanical characteristics as flexural strength and surface hardness. This method will not only help to reduce the failure that dentures are known to cause, but it will also capitalize on the ecological and the possible treatment advantages of natural materials. Until now, there is no systematic review that has analysed the outcome of incorporating plant fibers in PMMA denture resin and their impact on the mechanical properties of PMMA. Therefore, this systematic review aimed to explore the impact of plant fiber integration on the mechanical properties of PMMA denture base resins.

## Materials and methods

### Research question

Using the PICO model (Participants, Intervention, Control, and Outcomes), as outlined in the PRISMA guidelines, the subsequent research question was developed: ‘Does incorporating plant fibers in PMMA denture resins improve their mechanical strength?’ [41].

### Search strategy

Google Scholar, PubMed, and Science Direct databases were last searched on August 20, 2024. Journals related to dentistry and material science were examined electronically, and data were collected for a more comprehensive analysis.

### Inclusion criteria

The study includes full-text English-language articles published between 2014 and 2024 that addressed enhancing the mechanical properties of PMMA denture resin using natural fibers.

### Exclusion criteria

The study excluded articles that did not concentrate on using plant fibers to enhance the mechanical characteristics of PMMA denture resins. Articles published before 2014, written in non-English languages, or lacking full text, as well as reviews, meta-analyses, editorial letters, and ongoing research, were excluded.

### Literature search

**Figure 1:**
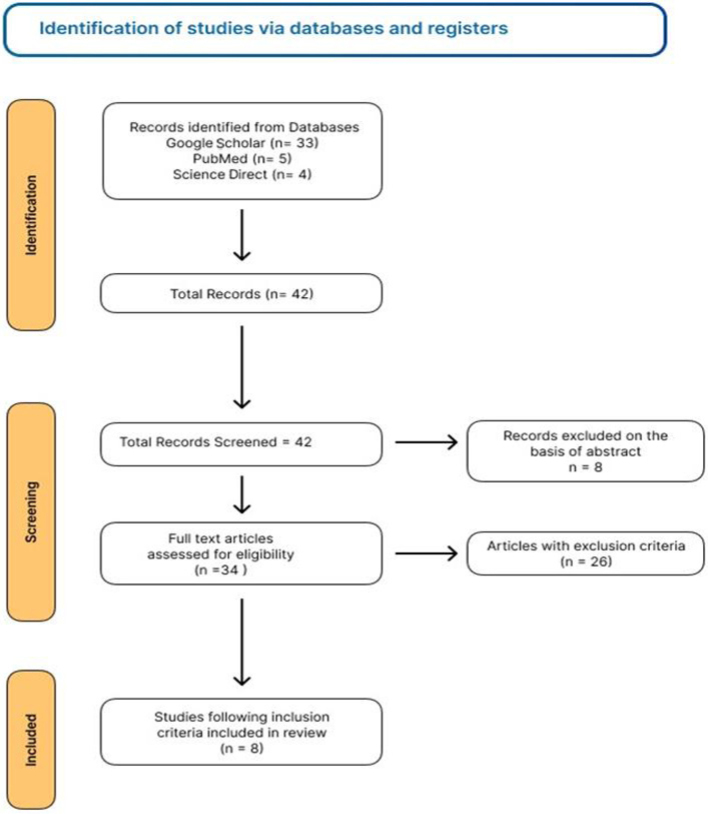
PRISMA flow diagram summarizing the study selection process for this systematic review.

A systematic search was conducted using well-known electronic resources, including ScienceDirect, Google Scholar, and PubMed, by two investigators (MAK and NM). Keywords ‘(PMMA denture resins) AND (properties) AND (fibers)’ were used for PubMed, ‘PMMA denture resins AND properties AND fibers’ for Science Direct, and ‘natural fibers and PMMA AND mechanical properties’ for Google Scholar, to retrieve the relevant articles. For this systematic review, only English-language studies published in the recent 10 years (2014–2024) were included, highlighted, and selected by two other researchers (SMM and BS). Furthermore, with the assistance of another pair of investigators (MKHU and MSZ), the reference list of articles was cross-checked. A total of 42 articles were screened initially. A total of 34 publications were excluded from the study; eight were rejected solely on the basis of their abstracts, and the remaining 26 were removed after reading the entire text. Figure 1 presents the detailed PRISMA flow chart indicating the process of identification, selection, screening, inclusion and exclusion of the research articles.

### Risk of bias

Two independent reviewers assessed the methodological quality of each included study using a modified risk-of-bias framework adapted from previously published systematic analyses for experimental laboratory [42, 43]. Method of sample preparation, sample count, random allocation, sample concealment, sample power calculation, operator blinding, International Organization for Standardization/American Dental Association standardized protocols, and experimental outcome were used as the basis for the guidelines used to evaluate the potential of bias. The criteria were given a score of ‘0’ if it was written clearly in the study. A score of ‘1’ was assigned if the necessary information was ambiguous or unclear, while a score of ‘2’ was given if a particular strategy was kept undisclosed. The study was considered to have a low tendency of bias if it had a score of 0–4, a moderate likelihood of bias if it received a score of 5–9, and a high potential of bias if it received a score of 10–14.

## Results

### General characteristics

All eight studies included in this review were experimental in vitro studies aiming to examine and evaluate the mechanical strength of denture base resins after incorporating different plant fibers. Mechanical properties such as flexural strength, impact strength, and transverse strength were observed with respect to denture base resin after incorporating plant fibers into denture base resins. Various plant fibers derived from *Hibiscus sabdariffa* [17]*,* banana [44], siwak [45], bamboo [45], sisal [46], and nano fibers derived from wood [47] and nanocellulose crystals (NCC) derived from bamboo fibers [48] were incorporated in denture base resins.

### Outcomes

Plant fibers extracted from *Hibiscus sabdariffa* incorporated in denture base resins resulted in enhanced flexural and impact strength as opposed to conventional acrylic denture base resins. Thus, denture resins reinforced with 7.5 wt% fibers revealed the maximum flexural strength (101.20 ± 3.65 MPa), followed by a fiber content of 10 wt% (88.67 ± 13.75 MPa), unreinforced (83.55 ± 0.62 MPa), 5 wt% (75.58 ± 3.68 MPa), and, finally, a fiber content of 2.5 wt% (68.84 ± 4.29) MPa [17]. Incorporation of sisal fibers with monomers in repairing denture base resins resulted in improved transverse strength of the acrylic denture base resins, as contrasted to those which were treated either only with sisal fibers or monomers [46]. Fibers extracted from washed and unwashed peels of banana incorporated in acrylic denture base resins showed enhanced flexural strength, tensile strength, impact strength, fracture toughness, and surface hardness of fiber-incorporated acrylic denture base resins as compared to traditional acrylic denture base resins [44]. Fibers reinforced at values of 5 and 10% improved mechanical properties compared to fibers reinforced at values of 15 and 20% [44]. An experimental study was conducted to evaluate the outcome of changing content and length of fibers (bamboo, miswak) on the mechanical properties of denture base [45]. The study found an increase in compressive strength when the content of fibers was increased, but a decrease in impact strength. In contrast, increasing the length of fibers, improved strength while compressive strength was decreased [45]. PMMA denture base material integrated with cellulose nanofibers (CNF) extracted from wood fibers exhibited significantly higher flexural strength in specimens reinforced with CNF, compared to pure PMMA, with the highest value achieved at a CNF concentration of 23% wt [49]. The incorporation of microcrystalline cellulose (MCC) derived from wood or cotton into acrylic denture base resins resulted in improved flexural strength, surface hardness, and surface roughness [50], with the highest flexural strength observed at a concentration of 5 wt% [47]. NCC derived from bamboo fiber resulted in significant increase in flexural strength when incorporated into denture base resins, compared to conventional PMMA, with the highest value achieved at a 0.5% wt. concentration [48]. Table 1 summarizes the included in vitro experimental studies that have attempted to improve the mechanical characteristics of PMMA by incorporating plant fibers as per PICO guidelines.

**Table 1:** Summary of the in vitro experimental studies that have attempted to improve the mechanical characteristics of PMMA by incorporating plant fibers as per PICO guidelines.

S.No	Author name and year	Study design	Population	Intervention (plant fiber used)	Comparison	Outcomes
1	Okeke et al. 2018 [17]	Experimental study	Laboratory-fabricated samples of PMMA denture base resins	*Hibiscus sabdariffa* fibers	Unreinforced conventional denture base resin PMMA	PMMA was tested for impact and flexural strength after being reinforced with hibiscus fibers with varying weight percentages (2.5, 5, 7.5, and 10). The specimens reinforced with 7.5 weight percent fiber had the significantly highest mean flexural strength (101.2 MPa) and impact strength (32 ± 0.015 MPa) when compared to the control groups.
2	Maharani AS et al. 2021 [46]	Experimental study	Laboratory-fabricated samples of PMMA denture base resins with denture repair section	Sisal fibers	Three groups were compared: Group I – repaired section treated with monomer; Group II – repaired section reinforced with fibers only; and Group III – repaired section treated with both monomer and fibers.	Highest transverse strength was observed in denture base treated with monomer and sisal fibers (133.45 ± 8.38 MPa), followed by denture base treated with sisal fibers alone (113.65 ± 7.31 MPa).
3	Khalil BL et al. 2017 [44]	Experimental Study	Laboratory-fabricated samples of PMMA denture base resins	Banana fibers	Unreinforced conventional denture base resin PMMA	Hardness was increased in acrylic base resin which was reinforced with 5% volume fraction of washed and unwashed banana peels (82 and 88 and 10% volume fraction of washed and unwashed banana peels (80 and 91) compared to unreinforced PMMA. Impact strength and tensile strength were found to be decrease in acrylic denture base resins reinforced with banana fibers.
4	Oleiwi et al. 2018 [45]	Experimental study	Laboratory-fabricated composite specimen of PMMA reinforced with siwak and bamboo fibers with varying concentrations (3, 6 & 9 wt%) and length (2, 6 & 12 mm)	Siwak and bamboo fibers with varying concentrations and length	Unreinforced conventional denture base resin PMMA	By increasing the weight % of fibers in PMMA, compressive strength increased while impact strength decreased. However, PMMA resin had improved impact strength but decreased compressive strength when the fiber length was increased. Compressive strength (530 MPa) was higher in the sample reinforced with siwak and bamboo fibers at 9 wt% and fiber length (2 mm)
5	Kawaguchi et al. 2020 [49]	Experimental study	Laboratory-fabricated samples of thermoplastic PMMA denture base material	CNF	Unreinforced conventional denture base resin PMMA	The flexural strength and modulus of reinforced samples were considerably increased by the addition of CNF. Flexural strength was highest in CNF-23 (96.8 ± 4.0 MPa), with progressively lower values in CNF-15, CNF-10, CNF-5 and CNF-0. Flexural modulus was also found to be highest in the CNF-23 group (3.96 ± 0.08 GPa).
6	Zaidan NM et al. 2022 [50]	Experimental study	Laboratory-fabricated samples of PMMA denture base resins	MCC	Conventional, unreinforced heat cure PMMA	The addition of MCC (silane treated) significantly enhanced and improved impact strength, surface hardness, and roughness.
7	Krishnasuthan et al. 2020 [47]	Experimental study	Laboratory-fabricated samples of PMMA denture base resins	MCC	Conventional, unreinforced PMMA	Flexural strength values for the control, 5 wt% MCC, and 10 wt% MCC groups were 40.5 ± 9.5 MPa, 57.4 ± 8.5 MPa, and 49.9 ± 5.6 MPa, respectively. The 5 wt% MCC group demonstrated higher flexural strength than other groups, with statistically significant results.
8	Aupaphong et al. 2022 [48]	Experimental study	Laboratory-fabricated samples of thermoplastic PMMA denture base material	NCC extracted from cellulose-rich bamboo fibers	Convention, unreinforced PMMA	NCC, derived from bamboo fibers, was used as reinforcement for acrylic resins at w/w percentages of 0, 0.25, 0.5, 1, and 2%. The mean flexural strength values of the five groups were 60.11 ± 2.4 MPa, 60.75 ± 2.18 MPa, 66.50 ± 5.08 MPa, 56.04 ± 0.31 MPa, and 48.05 ± 2.61 MPa. 0.5 wt. % reinforced group showed the highest flexural strength and 2 wt% the lowest.

PMMA: polymethyl methacrylate; PICO: Participants, Intervention, Control, and Outcomes; CNF: cellulose nanofibers; MCC: microcrystalline cellulose; NCC: nanocellulose crystals.

### Quality assessment

Seven of the included studies showed a minimum tendency towards bias, while only a single article was found to have moderate potential for bias. No article gave precise details about the blinding of the operator. Only one article mentioned a sample power calculation [48]. Two articles did not mention or give precise information on the sample count [44, 45]. All the studies mentioned the method of sample preparation, the random allocation of samples, standardized testing protocols, and experimental findings, respectively. Table 2 shows the risk of bias findings for all studies considered part of the systematic review.

**Table 2:** Risk of bias tool adopted from Sarkis et al. [42, 43]

Reference	Method of sample preparation	Sample count	Random allocation of sample	Sample power calculation	Standardized testing protocols	Blinding of operator	Experimental findings	Risk of bias
Okeke et al. [17]	0	0	0	2	0	1	0	Low
Maharani et al. [46]	0	0	0	2	0	1	0	Low
Khalil [44]	0	2	0	2	0	1	0	Moderate
Oleiwi et al. [45]	0	1	0	2	0	1	0	Low
Kawaguchi et al. [49]	0	0	0	2	0	1	0	Low
Zaidan and Jassim [50]	0	0	0	2	0	1	0	Low
Krishnasuthan et al. [47]	0	0	0	2	0	1	0	Low
Aupaphong et al. [48]	0	0	0	0	0	1	0	Low

## Discussion

To conduct this systematic review, the following research question was developed following the PRISMA protocol: Does incorporating plant fibers in PMMA denture resins improve their mechanical strength? Keywords with Boolean operators were formulated, and three databases – PubMed, ScienceDirect, and Google Scholar – were screened for relevant articles. A total of 42 articles were retrieved from these databases; only eight met the inclusion criteria and were included in the study. In addition, a comprehensive risk of bias evaluation was performed for all selected studies to assess potential bias arising from experimental design and methodological reporting. All studies presented a low risk of bias except one, which showed a moderate risk, and none showed a high risk of bias. Studies showed a clear trend in evaluating the flexural strength of reinforced resins. In this review, only a few studies surface-treated the fibers before incorporating them into denture resin, demonstrating a significant impact on the material’s qualities, as the treated fibers yielded superior results in resin than the untreated ones. Our analysis showed that the flexural strength and surface hardness of denture resins can be significantly increased by adding natural fiber reinforcement. Still, the data was inconclusive for the other mechanical properties (impact, tensile, and compressive strength).

Johnston et al. [51], in their study reported that impact pressures and flexural fatigue are the two primary causes of 68% of resin-based dentures fracturing after a few years of use. Flexural fatigue occurs when a denture base resin is repeatedly loaded and flexed, leading to structural failure due to the formation of microcracks at points of high concentration. These microcracks gradually merge over time, eventually leading to fracture [51]. Impact failure is caused by an unexpected drop or decisive blow or impact to the denture outside the mouth [51]. While impact failure accounts for 80% of fractures in mandibular dentures, fatigue and effects together account for most of the fractures in maxillary dentures [52, 53]. So, the ability of the denture-based material to sustain multidirectional and complex masticatory or accidental forces is deemed a prime requirement, and the denture base’s clinical lifespan is mainly dependent on its mechanical characteristics. Therefore, to overcome this clinical setback or failure, various methods have been utilized, and the reinforcing PMMA resins with natural fibers to improve their mechanical characteristics is an approach that has received substantial attention due to the fiber’s enhanced biocompatibility, renewability, and biodegradability [54, 55]. This review is the first to systematically evaluate the mechanical effect of fiber incorporation in denture resins by thoroughly assessing all related papers.

In the quality assessment of the included experimental studies, all but one was judged to have a low risk of bias. Confounding and measurement biases were observed across all studies since the samples were examined without the operator being blinded. Except for one study [48], no study quoted the sample power calculation. Other parameters of the quality assessment, including testing standards, sample fabrication technique, sample dimensions, sample randomization, and study findings, were all mentioned. Moreover, the low risk of bias indicates that a study’s methodology has minimal error or flaws, and that the findings can be considered reliable ; hence, the conclusions drawn in this review can also be considered credible and valid.

The foundation for strengthening PMMA by incorporating fibers rests on the theory that shear forces at the polymer-fiber interface enable the polymer matrix to effectively transmit an externally applied force to the fibers [56]. Hence, the main force-bearing components are the fibers used as reinforcing agents and encircled by a continuous phase of the matrix that retains them in place [56]. For this reason, fibers are required to be stiff to yield a noticeable strengthening effect on PMMA’s mechanical characteristics. Additionally, a satisfactory bond between the polymer matrix and fiber is crucial for the effective transfer of forces [57]. That is why fibers treated with coupling agents have the potential to have better interface bonding with the matrix and, hence, produce better clinical results. Two of the eight experimental studies included in our review, that incorporated treated plant fibers were conducted by Zaidan et al. [50], and Oleiwi et al. [45]. In the study of Zaidan’ et al. the MCC was treated with silane before it was added to the PMMA resins, whereas in the study by Oleiwi et al. and the bamboo and siwak fibers used for resin reinforcement were treating them alkaline solution of sodium hydroxide (NaOH). Both studies demonstrated improved mechanical properties when using these treated fibers. However, the results cannot be directly compared with other studies that used untreated fibers, as those studies used different fiber contents, concentrations, and lengths. Additionally, variations in methodologies and testing durations further complicate the comparison.

Clinically, midline fracture of the denture base is the most frequent fracture associated with PMMA-based resin, as Darbar et al., reported that midline fractures account for about 29% of all denture repair cases [57]. Therefore, evaluating the resin’s flexural strength is highly relevant, as this shows how well the resin can withstand fracture in reaction to bending forces. Five of the included studies conducted a bending test (3-point bending test) using a universal testing machine to measure the flexural strength of reinforced resins, reflecting methodological consistency across the studies. All included studies found that the reinforced PMMA showed significantly increased flexural strength as compared to control samples. Also, data demonstrated that the flexural strength of the reinforced resins increases with increasing fiber content; however, the concentration showed a threshold effect on the flexural strength. Once a concentration reached a certain threshold, further increases in fiber content did not enhance the strength. Aupaphong et al. [48], incorporated NCC in PMMA at four different concentrations, which were 0.25, 0.5, 1, and 2%. Findings revealed that the PMMA resins’ flexural strength increased with fiber concentration up to 1% and then declined at 2%. Krishnasuthan et al. [47], in their study, also utilized two weight concentrations (5 and 10%) of MCC in PMMA resins and noted that 5% showed higher flexural strength than dig 10%. In their in vitro study Maharani et al. [46], incorporated sisal fibers during denture repair and assessed its flexural strength, and reported that the flexural strength of the restored dentures was enhanced by the addition of fibers.

Only three studies evaluated the impact strength of PMMA, and all three utilized Charpy’s impact testing device [17, 45, 50]. Two of these studies demonstrated that increasing the fiber concentration can effectively enhance the PMMA impact strength [17, 45]. On the other hand, one study [45], which determined the impact strength using various concentrations and lengths of natural fibers (siwak and bamboo), reported that the impact strength of the resins improved with longer fiber lengths but declined as the fiber weight concentration increased. This result contrasts with the results of the other two studies. The same study also evaluated the compressive strength of the reinforced resins, showing that it increased with increasing fiber weight percentage. However, no other studies assessed the compressive strength after reinforcement.

Another critical mechanical characteristic that was assessed by only two of the included studies was surface hardness [44]. Studies used a Shore D durometer to evaluate hardness. Zaidan et al. [50] demonstrated that the reinforced resins (1 and 1.5% MCC) exhibited greater surface hardness compared to the control. Khalil et al. [44] also evaluated the surface hardness of resins reinforced with washed and unwashed banana peel at 5, 10, 15, and 20%. The 10 wt% group showed the highest surface hardness, and washed banana peel yielded better results than unwashed peel. This study also investigated the tensile strength and found that the addition of fiber reduced the acrylic resin’s tensile strength.

Almost all the included studies followed the same methodology, which makes the results comparable. Collectively, the evidence indicates that plant fiber–reinforced PMMA exhibits superior flexural strength and surface hardness compared to conventional PMMA. Notably, among all the studies reviewed, the PMMA formulation incorporating *Hibiscus sabdariffa* fiber at 7.5 wt% demonstrated the highest flexural strength , outperforming all other plant fiber–reinforced PMMA compositions reported in the literature [17]. For surface hardness, the PMMA reinforced with 10% washed banana fibers demonstrated the highest hardness value, exceeding that of all other fiber-reinforced formulations reported in the included studies [44].

The main strength of this systematic review is that it is the first review that has investigated the effect of reinforcing natural plant fibers on the mechanical properties of PMMA, and all the studies included in this review showed a very low risk of bias, which made its results credible and comparable. The primary limitation of this systematic review is that all eight studies included used different types of plant fiber incorporated into PMMA resin. This resulted in significant heterogeneity, as the included studies varied greatly in terms of the type and source of plant fibers, their particle size, concentration, and surface treatment methods. Such material-related differences make direct comparison challenging and may limit the ability to draw uniform or generalized conclusions from the available evidence. Additionally, a limitation of this study is that none of the studies evaluated the properties of fiber-reinforced PMMA under a wet storage regimen. The absence of testing in conditions that mimic the humidity of the oral environment may compromise the clinical relevance of the findings. It could lead to potential misinterpretation of the materials´ actual performance in vivo. Considering these limitations, it is evident that very few studies have explored the incorporation of natural plant-based fibers into PMMA to enhance its mechanical performance. There is a significant scarcity of long-term experimental studies and clinical trials in this area. Consequently, it is essential to conduct long-term experimental studies, especially under both dry and wet storage conditions, as well as clinical trials, to more accurately evaluate the performance and longevity of plant fiber-reinforced PMMA in the oral cavity.

## Conclusion

The integration of natural fibers into acrylic resin formulations significantly improves both flexural strength and hardness. However, the data were found insufficient and inconclusive for other mechanical properties, including impact, compressive, and tensile strength. Therefore, to further ascertain its clinical application and longevity, long-term experimental studies involving aging and varying storage regimes, as well as clinical trials, are required.

## Data Availability

The data will be made available as per requirement.
